# Enamel and Bleaching or Breaching: Vickers Hardness and Backscattered Electron Imaging

**DOI:** 10.1007/s00223-026-01518-6

**Published:** 2026-04-01

**Authors:** Alan Boyde, Shima Ashrafianbonab, David Mills

**Affiliations:** Dental Physical Sciences Unit, Institute of Dentistry, Queen Mary University of London, Mile End campus, London, E1 4NS UK

**Keywords:** Enamel structure, Etching, Demineralisation, Peroxide, Microscopy, Adverse effects, Porosity

## Abstract

Although healthy teeth are never really white, bleaching agents are used cosmetically to whiten teeth. We studied the in vitro effects of some bleaching agents and acids on the Vickers Hardness (VH) of human dental enamel using a 100 gram [981 mN] load applied for 15 s and examined treated surfaces with backscattered electron mode scanning electron microscopy (BSE SEM; 20 kV, uncoated, chamber pressure 50 Pa). Rows of indents were made in the untreated condition and intervening rows placed after treatment. Thus, the samples served as their own controls. Gel bleaching agents − 35% hydrogen peroxide or carbamide peroxide - and 30% hydrogen peroxide solutions caused substantial reductions in microhardness. Weaker peroxide gels and solutions caused lesser changes. A 5% available chlorine sodium hypochlorite bleach treatment resulted in no significant change. Prism boundary discontinuities in flat polished surfaces are normally thin and dark in BSE-SEM imaging because of the lower mineral content in the region where crystallite orientation changes abruptly – they are also not joined up. Strong peroxide bleached surfaces, however, showed wider, whiter and conjoined boundaries as in acetic acid treated samples. This appearance is the hallmark of subsurface demineralisation and remineralisation, wherein the boundary region is widened and extended by dissolution of mineral, with reprecipitation of dissolved mineral in surrounding partially demineralised tissue and infilling of the original boundary defect. Identical sequences are identified in natural and artificial carious lesions and in acid etched enamel. This appearance was not generated by 6% peroxide gel. The softening of enamel by peroxide bleaching agents is explained both by the removal and redistribution of the hard mineral phase, accounted for by their acidic (i.e. low pH) nature; and the removal of the enamel protein matrix ‘glue’ holding the crystallites together.

**Supplementary Information:**

The online version contains supplementary material available at 10.1007/s00223-026-01518-6.

## Introduction

Enamel has two main components, mineral and organic matrix, both of which may be altered, degraded or dissolved by reagents which are used to treat the tissue, either in the patient or during the conduct of research in the laboratory. Here, we were initially concerned with agents which are intended to bleach, rather than to etch enamel. However, peroxide solutions are acidic, the pH decreasing with increasing concentration.

There is a large literature on bleaching and hardness. Many prior studies have reported deleterious effects of peroxide bleaching agents on enamel, including softening, i.e. a measured reduction in the indentation microhardness, or demineralisation as in incipient subsurface reduction in mineral content seen in natural and artificial caries [26, 33, 14, 15, 19, 20, 21, 22, 40, 41, 45, 48]. 

Others have reported no significant change [29, 47,  24, 23, 34, 42], and a few have concluded that the enamel is harder after treatment [2, 38]. 

However, the consensus seems to be that peroxide bleaches do soften enamel and many studies have been directed towards – and have been at least partially successful - in preventing or reversing decalcification by adding something during or after the treatment, such as by making the bleach solution neutral or alkaline, or by adding fluoride, Bioglass, or finely divided calcium phosphates [26, 28, 5, 16, 17, 18, 27, 30, 32, 35, 36, 37, 39, 44, 31].

Others have stated that a natural recovery from the negative consequences of peroxide bleaching happens in vivo due to the acquisition of mineral and/or organic components from saliva and suggest that in vitro experiments alone may give a wrong impression [4, 46].

As regards the types and origins of samples used for laboratory experiments, some investigators have used natural tooth surfaces, with the problem that micro-anatomical details of the enamel surface [residual Tomes’ process pits, imbrication lines and perikymata] vary greatly from place to place on one tooth and from tooth to tooth. Less variability is found in the subsurface enamel that can be exposed by polishing flat surface facets, which are in any event better for microhardness testing.

Many studies have used bovine incisor teeth even though the enamel development and structure are radically different from human. Bovine enamel is Pattern 2, with inter-row sheets of interprismatic enamel, whereas human enamel is Pattern 3 and mostly cannot be seen to have any ‘interprismatic’ enamel as it is all in ‘prisms’ [6, 8]: bovine enamel is also less mature, i.e. it retains a greater fraction of enamel organic matrix proteins.

In the laboratory, we use sodium hypochlorite solutions to clean enamel surfaces prior to scanning electron microscopy. The same treatment renders *dentine and cementum* ‘anorganic’ – their organic matrix components are rapidly degraded and dissolved to expose their mineralising fronts [10], and these tissues may be so weakened internally that a tooth will break between finger and thumb. Prolonged hypochlorite treatment may also weaken *enamel* – of advantage if one wants to break the tissue to study its internal structure – but this is in large part due to extreme softening of the underlying deproteinised dentine which can be washed away from the enamel with a water jet [8].

In endodontic therapy, dilute hypochlorite or peroxide solutions are used to sterilise root canals, when the liquid is contained within the tooth. For external use during tooth ‘whitening’ in the clinic, concentrated hydrogen peroxide (HP) or carbamide peroxide (CP) are used in gel formulations to restrain fluid flow.

The whiteness of a tooth depends on light scattering from minute imperfections in enamel such as the prism boundary discontinuities. The more perfect the mineralisation of enamel, the more it is translucent allowing the yellow colour of dentine to show through. A newly erupted permanent tooth is whiter than a tooth that has erupted for a long time: post-eruptive maturation makes enamel more translucent, less white: this normal change is beneficial. Poorly mineralised enamel is whiter: early carious lesions are white. Whiteness is therefore not necessarily a virtue in enamel.

The purpose of the present study was to develop a background for future protocols to characterise damage done during clinical and/or laboratory ‘bleaching’.

## Materials and Methods

### Human Tooth Samples

We used apparently sound human teeth stored in 70% ethanol, from an anonymised collection made before the Human Tissue Act 2004: there were therefore also no ethical issues concerning the use of this material. We tried to maximise the number of test samples obtained from each tooth.

Approach 1 was to prepare plane parallel longitudinal sections (LS) which also permitted histological study. Seven mesio-distal sections were prepared from an upper third permanent molar (UR8A) cut using a Buehler low-speed water-cooled diamond saw. One surface of each section was finished using 1200 and 4000 grit silicon carbide abrasive paper flushed with running water, washed, briefly dried, and attached to a glass light microscope (LM) slide using cyanoacrylate superglue: the free section surface to be used for testing was then similarly finished on the wet abrasive papers, incidentally, removing any surplus glue. However, such LSs do not simulate the direction of attack of a bleaching agent perpendicular to a natural tooth surface, although having the advantage of sampling the enamel to all depths.

Approach 2 was to cut four faces from the tooth freehand by using a platform on the Buehler saw, avoiding the need to clamp the tooth during cutting. The cut surfaces of the resulting mesial, lingual or palatal, distal and buccal slabs were flattened with wet abrasion, briefly dried, and attached to the LM slide using cyanoacrylate. Then a flat facet, parallel with the slide, was prepared by polishing an area of the free natural surface of the tooth. In retrospect, this was the simplest and most rapid method for getting robust samples.

Approach 3 was to cut the tooth [secured during cutting with Kerr’s greenstick impression compound] using an Exakt water cooled bandsaw, first mesio-distally and then each half again buco-lingually to produce four corner samples [e.g. disto-buccal]. The rectangular inner corners were flattened against coarse abrasive paper before glueing to the slide with rapid setting epoxy glue. Surface parallel facets were polished as above.

All samples were kept damp, stored in plastic Petri dishes with a wad of wet paper tissue in a 4 °C refrigerator, except when being tested or imaged.

### Buehler Isomet

We used a commercial microhardness tester built into a reflected light microscope (LM) frame [Buehler Isomet] to make the indentations used to determine the Vickers Hardness (VH) values of enamel This apparatus provided a clamping mechanism for standard 25 mm wide glass LM slides. We practiced with indents made in glass slides - which we assumed to be a uniform material - so that we could develop a sense for the reliability and reproducibility of the method (Suppl. Figures 1–3).

**Fig. 1:**
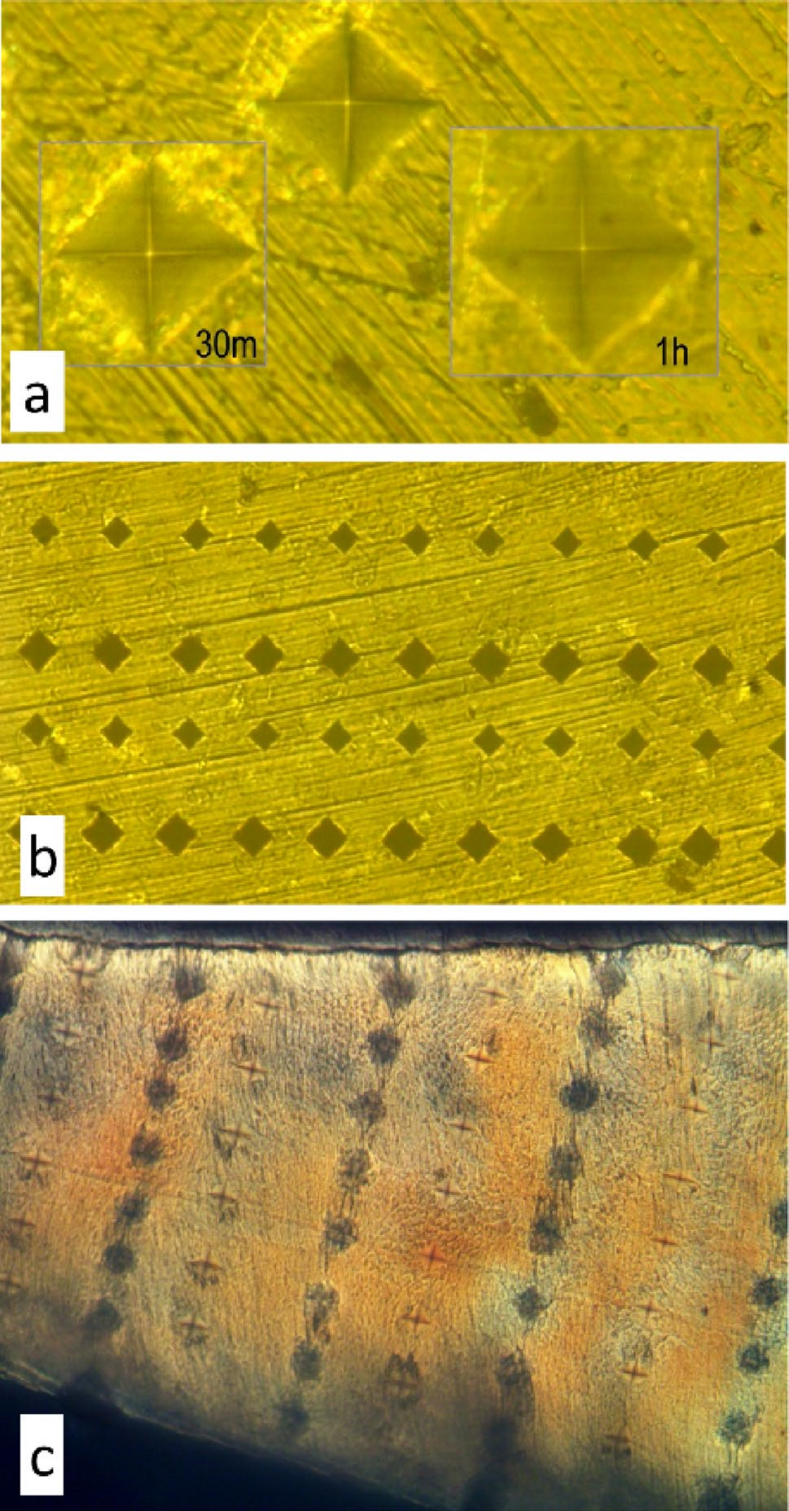
Light microscopic images **a**. Reflected light images of microhardness indents from digital camera on Buehler Isomet hardness tester: before [background image field width 104 μm] and after treatment with 35% HP gel for 30 m [dark blue outlined inset left] and 60 min [inset right]. Upper third molar, longitudinal section. **b**. Lower magnification reflected light image of microhardness indent rows, smaller before and larger after treatment with 35% HP gel for 5 h. upper third molar, distal surface slab. Field width 513 μm. **c**. Transmitted circularly polarised light image of lateral enamel in longitudinal section of upper third molar, showing damage zones [dark] around the pre-treatment rows of indents by 35% hydrogen peroxide gel bleaching. Field width = 570 μm. The same field is shown as a through focus stack in Suppl. Figs

The Isomet system was equipped with a digital camera, and rather than using the eyepiece graticule measuring device with the need to rotate the device through 90° for each indent, we made the necessary measurements of the diagonals of the indents *offline* – using digital images recorded with a 50/0.68 objective - to speed the process (Fig. 1a, b). We calibrated the image pixel size using a silicon wafer standard: this differed by a few percent from the values given by the system. We confirmed the load delivered by the system using a 3-axis load cell and standard calibration weights.

We used a 100-gram load with the standard 136° faceted diamond indenter tip for all enamel measurements applied for 15 s. [A 100 gram load was too high for dentine where 25 g produced indents a little bigger than the 100 g in enamel]. Different parts of enamel surface may have different hardness values. Indents were made in rows at 50 to 60 μm spacing, leaving 100 to 120 μm gaps between rows to interpose more indent rows in the same locality after treatment. Therefore, all the indents, before and after bleaching, were made in the same areas ensuring that the before and after treatment measurements used similar tissue.

As we always used the same load and the same objective lens and camera, we could estimate effects merely by looking at the images or the measured indent diameters in raw pixel values before for converting the numbers to VH.

Vickers Hardness (VH) was calculated from VH = 1.891 F/d^2^, where F is in kgf and d is in millimetres.

### Optical Microscopy of the Longitudinal Section Samples

In this study ordinary transmitted light microscopy and circularly polarised light (CPL) were used to take and record images of the section samples: (a) after sectioning the tooth and mounting of the slides (before making indentations): (b) after making indentations on the specimens (before bleaching): and (c) after bleaching and making additional indentations on the specimens. Images were recorded using a finite tube-length optical microscope using 2/0.08, 4/0.2, 10/0.45 and 20/0.75 objectives, in ordinary transmitted light linearly polarised light with crossed polarising filters (XPL, PLM) and with circularly polarised light (CPL: Fig. 1c), using an AmScope MU900 microscope digital camera and control software. Through focus stacks were processed using Syncroscopy (Cambridge) AutoMontage software and ImageJ.

### Optical Coherence Tomography

This technique uses low-coherence near-infrared light to produce high-resolution cross-sectional and three-dimensional images. Any areas of mineral loss and increased pore size in the enamel can be detected due to increased light scattering and reflectivity. OCT was used to take and record images of the section samples mounted on slides (Thor Labs Ganymede spectral domain 880 nm). This helped to make sure that all the samples were free of enamel defects, such as hypocalcification, cracks, or incipient carious lesions that might be undetectable by visual inspection. One sample showed some degrees of hypocalcification in the area close to the depth of the central fissure. However, this was of no concern as all the indentations were made in the cuspal and lateral regions of the enamel. There was no sign of any other enamel defects in the specimens.

Bleaching agents and other treatments.

**Table:** 

Bleaching agents	Treatment times
10% HP solution SIGMA	18, 20, 40 h
30% HP solution SIGMA	20, 60 min
POLA DAY 6% HP gel	18, 38, 104 h
LaserGlow 35% HP gel.	20, 30, 40, 60 min; 5, 20 h
POLA NIGHT 16% carbamide peroxide gel	18 h
5% NaOCl solution	40 h

We measured the pH of liquids and gels used in this study using a Mettler Toledo Seven2go pro S8 pH meter.

0.60: Icon Etch (DMG) gel, brand-name for a 15% hydrochloric acid gel.

0.95: Super etch (SDI) gel, brand-name for a 37% phosphoric acid gel.

2.50: 30% hydrogen peroxide solution packing date 18 months prior to use.

2.80: 30% hydrogen peroxide solution packing date – older stock.

2.97: 5% acetic acid.

3.01: 35% hydrogen peroxide gel.

4.00: 0.1 M sodium acetate buffer solution.

5.90: 10% hydrogen peroxide solution.

6.23: 6% hydrogen peroxide gel.

6.39: 16% carbamide peroxide gel.

7.58: Freshly distilled water.

7.66: Fresh tap water, having let the tap run for a few minutes.

12.5: 5% available chlorine, sodium hypochlorite solution.

### Comparison and Statistical Evaluation of Indent Sizes: Fig. 2a–d: Supplemental Statistical Output

**Fig. 2:**
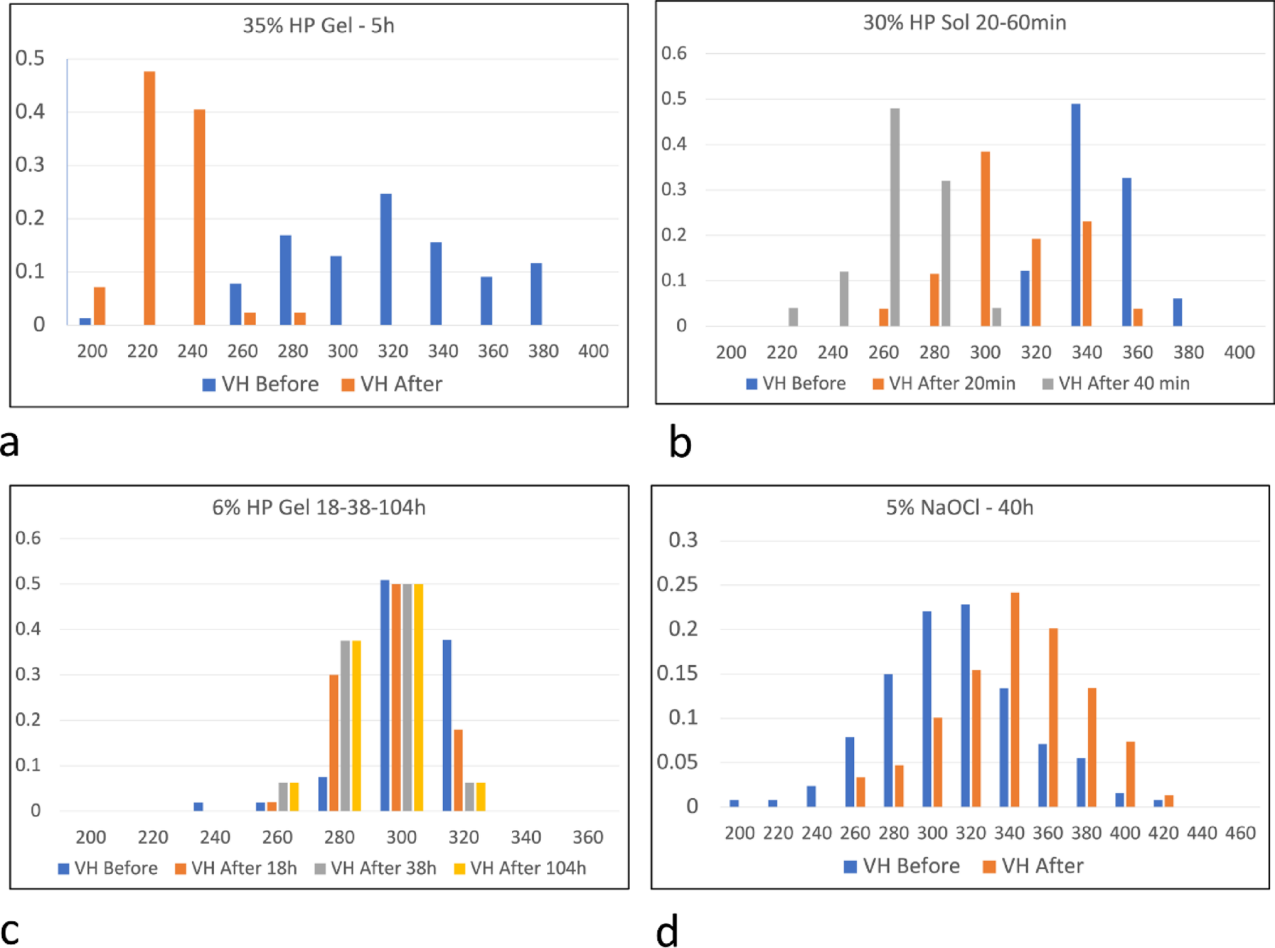
Histograms of Vickers hardness, vertical axes normalised frequency **a**. Vickers hardness (VH) before and after bleaching with 35% hydrogen peroxide gel for 5 h. Longitudinal section upper third molar. **b**. VH before and after bleaching with 30% hydrogen peroxide solution for 20 m and 60 m. Corner slab upper third molar. **c**. VH before and after treatment with 6% HP gel for 18 h, 38 h and 104 h. Distal face of upper third molar. **d**. VH before and after bleaching with 5% available chlorine sodium hypochlorite for 40 h. Longitudinal section upper third molar

We tested for statistically significant differences in enamel hardness between unbleached and bleached enamel with peroxide- or hypochlorite-based bleaches. 1-way ANOVA and paired sample t-tests were used for 35% HP Gel, 30% HP solution, 6% gel. Paired sample t-tests were used for 10% HP solution, 16% HP Gel and 5% NaOCl solution (*p* < < 0.05 in all cases) - see Fig. 2a–d and supplemental statistical output.

### Scanning Electron Microscopy (BSE SEM) of Indented Samples: Fig. 3a–f

**Fig. 3:**
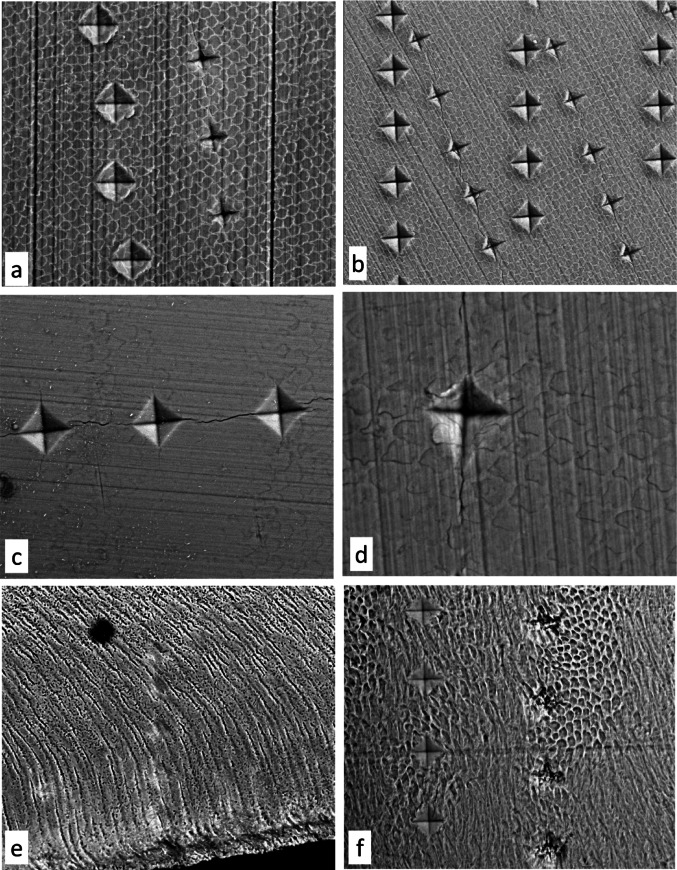
a–f 20 kV BSE-SEM, uncoated, indents: prism boundaries are white and continuous in **a** and **c**, evidence of subsurface demineralisation and remineralisation. **a.** Lingual surface of lower right third permanent molar. Parts of two rows of 100-gram indents made before (right) and after treatment with 10% hydrogen peroxide solution for 40 h. Field height = 179 μm. **b.** Upper third permanent molar, corner facet. Smaller Indents made before and larger after treatment with 35% hydrogen peroxide gel for 20 h. Field height = 254 μm. **c.** 100-gram load hardness indents in untreated buccal surface facet of upper third permanent molar. Note separate, dark prism boundary discontinuities reflecting their lower mineral concentration. Visibility of the prism boundaries varies with depth in the enamel corresponding to the subsurface developmental incremental subsurface layering [regular 8–9-day interval sub-surface striae of Retzius; perikymata; imbrication lines: see [8] ]. Field height = 118 μm. **d**. Slab facet sample, upper third permanent molar distal, treated for total 104 h with 6% hydrogen peroxide gel retains dark and separate prism boundary markings, indicating no significant demineralisation or remineralisation. Field height 71 μm. **e** All the bleaching regimes we used cause severe damage to dentine and cementum by removal of the collagenous organic matrix and possibly also some mineral. Here, a row of 25-gram indents in dentine is scarcely traceable after 10% hydrogen peroxide solution treatment. Dentine has separated from enamel at the EDJ [dark area, lower right]. Upper third molar, longitudinal section. Field height = 348 μm. **f** Same longitudinal section of upper third permanent molar treated with 10% hydrogen peroxide solution for 18 h, lateral enamel, showing etching of enamel to reveal HSBs [diazones and parazones]. Reference or baseline row to right shows damage to its indents by the ‘bleaching’. Field height = 194 μm

After the quantitative microhardness studies were completed, samples were allowed to dry before examination in the SEM (Fig. 3a–f). We used the uncoated samples still attached to the glass slides in a Zeiss EVO MA10 SEM in ‘vapour pressure’ mode with the chamber vacuum at 50 Pa. Images were recorded using backscattered electrons (BSE SEM) at 20 kV [10].

We also reprocessed stereo-pair images recorded with 10° tilt angle difference from an earlier enamel etching study, since modern computer displays – not available at that time - make appreciation of 3D vastly simpler and more effective: either as a flicker display showing the two members of a stereo-pair in quick succession or as anaglyphs to be viewed with red-cyan viewing filters on the display monitor ([10, 12]; Suppl. Figures 4 & 5). These images were recorded with a Cambridge Stereoscan S4-10 SEM using secondary electrons, 10 kV accelerating voltage from gold coated specimens.

### Additional SEM Samples and Treatments: Fig. 4a–d

**Fig. 4:**
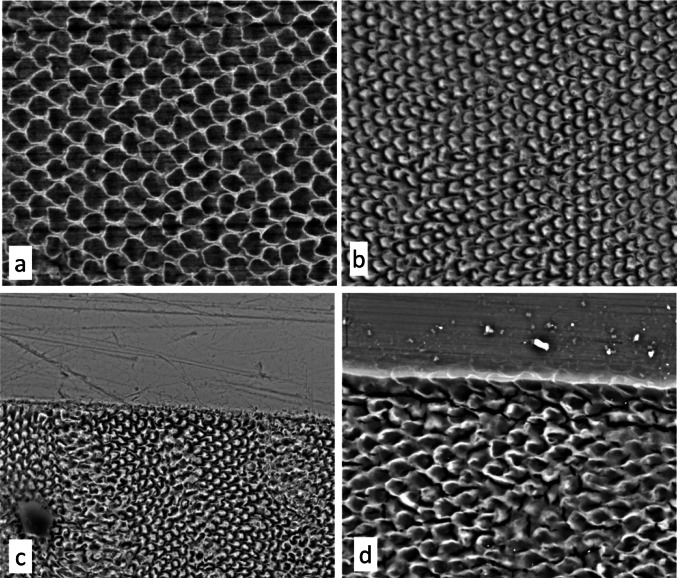
20 kV BSE-SEM, uncoated, enamel ‘etching’ **a** Lower first premolar, distal, polished surface facet, treated with 5% acetic acid for 2 h: surface has ‘etched’ to leave the white honeycomb typical of weak acid induced subsurface demineralisation and remineralisation. Field height = 84.6 μm. **b** Lower first premolar, distal, polished surface facet, etched with 15% hydrochloric acid gel [Icon Etch] for 2 min. Etching has removed tissue adjacent to the prism boundary discontinuity spaces thus expanding these spaces. Field height = 121 μm. **c** Labial surface of upper lateral incisor, natural polished surface partly covered with PMMA in chloroform ‘varnish’ now removed, etched with 37% phosphoric acid gel [DMG Super Etch gel] for 1 min. Etching has removed tissue adjacent to the prism boundary discontinuities except in the planes of the sub-surface incremental lines. Field height = 187 μm. **d** Upper third molar, buccal, polished surface facet, upper area protected with nail varnish now removed, treated with 30% hydrogen peroxide solution for 66 h, surface has eroded leaving etching pattern with continuous elevated honeycomb. Field width = 570 μm

After the quantitative microhardness studies were completed, we re-used some of the original samples and prepared more previously untreated samples to provide further comparative BSE-SEM insights on the effects of bleaching agents, weak acids, and clinically used gel etchants on surface and sub-surface enamel (Fig. 4a–d). Solutions or gels so tested included 5% acetic acid, a 0.1 M pH 4 acetate buffer, Icon Etch (brand-name for a 15% hydrochloric acid gel), Super Etch gel (SDI) which is a 37% phosphoric acid etchant, and a 30% hydrogen peroxide solution.

To protect parts of the enamel surface against attack we used either strands of a syrup of poly-methyl-methacrylate (PMMA) dissolved in chloroform or nail varnish (amyl acetate solvent). These were allowed to air dry before treatment and removed after treatment with acetone or chloroform. BSE-SEM imaging was conducted as above (Suppl. Figures 6–8. To illustrate height differences caused by etching, we used high tilt angle viewing or stereo-pair imaging by tilting the nearly flat sample between exposures, or [12].

### Number of Samples, Measurements and SEM Images

The total number of teeth used was 11. The number of sections or slabs mounted on glass slides was 28. The number of indentations and corresponding images recorded with the 50/0.68 objective and subsequently measured using ImageJ to calculate VH was 1855. The number of BSE-SEM images recorded after the indentations were completed was 617.

## Results

### Microhardness

In all experiments, we observed statistically significant differences in enamel hardness between unbleached and bleached enamel with peroxide- or hypochlorite-based bleaches. 1-way ANOVA and paired sample t-tests were used for 35% HP Gel, 30% HP solution, 6% gel. Paired sample t-tests were used for 10% HP solution, 16% HP Gel and 5% NaOCl solution (*p* < < 0.05 in all cases).

**Table:** 

Group	*N*	Mean (SD)
Baseline (LR8 – Mesial)	30	415.5 (15.9)
20 h 10% HP sol	22	223.3 (14.1)
Baseline (LR8 – Lingual)	22	415.3 (12.9)
40 h 10% HP sol	30	279.8(20.8)
Baseline (UR8B-Palatal)	47	414.2 (12.6)
20 min 35% HP gel	24	360.2 (12.3)
40 min 35% HP gel	24	351.4 (16.6)
Baseline (UR8A-Sec6)	51	303.1(25.3)
30 min HP 35% gel	22	262.1(21.3)
60 min HP 35% gel	28	204.8(21.2)
Baseline (UL8B-W)	51	398.0(12.0)
20 h 35% HP gel	37	199.7(13.4)
Baseline (UL8B-U)	49	395.8 (14.1)
30 min 30% HP sol	26	363.6 (23.8)
60 min 30% HP sol	25	314.5 (17.3)
Baseline (UR8B-Buccal)	51	412.2 (17.6)
18 h 16% CP gel	25	276.8 (30.2)
Baseline (UR8B-Distal)	53	393.8 (14.6)
18 h 6% HP gel	50	386.8 (13.6)
38 h 6% HP gel	16	381.6 (14.0)
104 h 6% HP gel	36	331.8 (16.2)
Baseline (UR8A-Sec4)	127	302.5 (36.5)
40 h 5% NaOCl sol	149	332.3 (34.7)
Baseline (UR8A-Sec3)	77	307.0 (37.7)
5 h 35% HP gel	41	219.6 (14.3)

All the peroxide agents tested reduced the hardness of the enamel - see Fig. 2a–c and the Supplemental Statistical Output file.

The only regime which did not soften the enamel was that using a 5% available chlorine sodium hypochlorite solution (Fig. 2d). This was the only reagent which has a high pH, i.e., is strongly alkaline.

### Extent of Indenting Damage

Transmitted LM and CPL studies of the *sections* showed that bleaching samples with had indents caused the tissue in and near those indents to fault (Fig. 1c: Suppl. Figure 9) or, in the case of dentine and cementum, to nearly disappear (Fig. 3e). This bleaching damage around baseline indents could also be seen in both sections and slabs with the reflected LM of the testing machine and afterwards by BSE-SEM study.

Both the reflection LM and BSE-SEM images of indents show cracking in enamel outside the immediate area of the indent and piling-up and displacement of material in the immediate surrounding area (Suppl. Figure 10). Likewise, CPL and PLM imaging of indents in glass slides showed cracking and induction of strain birefringence extending to great distances from the indent proper.

### BSE SEM Detail

In BSE-SEM imaging of polished enamel surfaces, prism boundary discontinuities are normally thin, separate and dark, indicating a lower mineral content (Fig. 3c: [8, 11]). Particularly in the higher peroxide concentration CP and HP gel bleached samples, the boundary discontinuities were whiter, indicating a relatively higher mineral content, wider, and conjoined, signifying the cervical expansion of discontinuity space (Fig. 3a, b). 6% hydrogen peroxide gel treatment left the prism boundaries in the normal state, i.e. narrow, dark and mostly discontinuous even after prolonged treatment (Fig. 3d).

Immersion in 5% acetic acid solutions (pH < 3) for 2 h and 19 h generated the same type of appearance seen with strong peroxide bleached samples (Fig. 4a). Treatment of enamel surfaces with the low strength pH 4 acetate buffer induced no detectable change in samples treated for 2 h up to 48 h. Interestingly, vinegar has been used as a home-based tooth bleach [49, 1] and Agarwal et al. [3] consider that the active agent in the ‘lowest rated over the counter’ bleaches is acetic acid!

The damage done by the 15% hydrochloric acid gel (Icon Etch: DMG) in 2 min in sub-surface enamel can be seen in Fig. 4b. Several microns of enamel are removed, and a relatively deep etch has occurred by the expansion of the prism boundary discontinuity space. Similarly, 60 s erosion with 37% phosphoric acid gel (Super Etch: SDI) removed several microns of enamel and expanded the prism boundary space: the etching is less clear in the planes of the immediately sub-surface, regular circaseptan incremental lines (Fig. 4c).

Examination of HP treated, partially protected samples treated for 66 h also showed the removal of several microns of enamel depth and the development of an etch pattern of a continuous honeycomb, which is characteristic of a process of prior reprecipitation of mineral in enamel surrounding the prism boundary space: i.e., the remaining enamel surface is not a part of the original enamel structure (Fig. 4d).

## Discussion

Elfallah and Swain, [21] concluded that the ‘considerable differences in opinion as to whether changes in mechanical properties [microhardness] occuring as a result of tooth whitening appear to be related to the load applied during the indentation tests. Most studies which used loads higher than 500 mN [51 grams] to determine enamel hardness showed no effect of bleaching, whereas those using lower loads were able to detect hardness reduction in the surface layer of enamel’. Thus, we express no surprise that Seghi and Denry [43] found no effect on microhardness when testing with a 1 kg [9807 mN] load, although they showed that apparent fracture toughness of enamel was reduced by about 30% after bleaching for a period of 12 h.

The load applied during microhardness testing is of such paramount importance because the depth of organic matrix destruction and demineralisation with peroxide bleaches is shallow. A high load will ‘test’ predominantly the deeper, unaffected tissue. The load is such an important parameter that it should be specified in the abstract text of any publication in this area.

The 100-gram load which we used was sufficiently sensitive to give the results which we did achieve. But if we chose to test less aggressive bleaching routines, we might be advised to choose a lower load, say the 50-gram setting on the testing machine we used. Here, however, we would have a problem with the precision of the measurements, since the magnification of the higher-powered LM objective would be insufficient. The solution would be to make all the measurements from BSE-SEM images, when we could choose any magnification and resolution at will.

The present results show that a standard form of sample preparation - in which human teeth sides or quarters are glued to LM slides, leaving natural faces exposed parallel to the slide which are then polished to generate flat subsurface facets - is well suited to use both in a standard VH testing machine and in an SEM. Large numbers of indents can be made in each sample, thereby reducing the number of teeth required and inter-sample variability, and the same samples can be used as their own controls by interleaving post-treatment rows between the control pre-treatment indents. Furthermore, by measuring from recorded images, off-line, we can make larger numbers of VH measurements to produce more reliable survey statistics.

As regards the types of sample preparation that were used, the initial ones were longitudinal sections, because it is easier to obtain many from a single tooth. VH values obtained from sections, both before and after different bleaching methods, showed wider standard deviations compared to the surface parallel slabs which better equate to worn and polished natural enamel surfaces. This can be explained by the fact that, unlike enamel prisms in the enamel surfaces, which are roughly perpendicular to the surface, the enamel prisms in the longitudinal sections have different directions in their course between the EDJ and the enamel surface [Hunter-Schreger bands (HSBs): diazones: parazones Fig. 3f]. These variations in the prism directions result in a surface of enamel with areas of differing microhardness [7, 8, 13, 25] . The natural-face-parallel samples were more consistent because the prism orientation is more consistent, i.e., decussation is less, in the subsurface zone of radial enamel.

However, an advantage of using sections rather than slabs is that the extent of damage around indents caused by bleaching can be easily spotted with transmitted LM. That this occurs is an interesting phenomenon, because it suggests that bleaching would predispose to enhanced tissue loss during the natural indenting occurring during normal wear and tear in vivo. Local impacts on the enamel surface due to harder food particles would cause more local damage to bleached enamel. The consequences of bleaching – or breaching – are that enamel is more easily damaged thereafter.

### Etching and Redistribution of Mineral

**Fig. 5:**
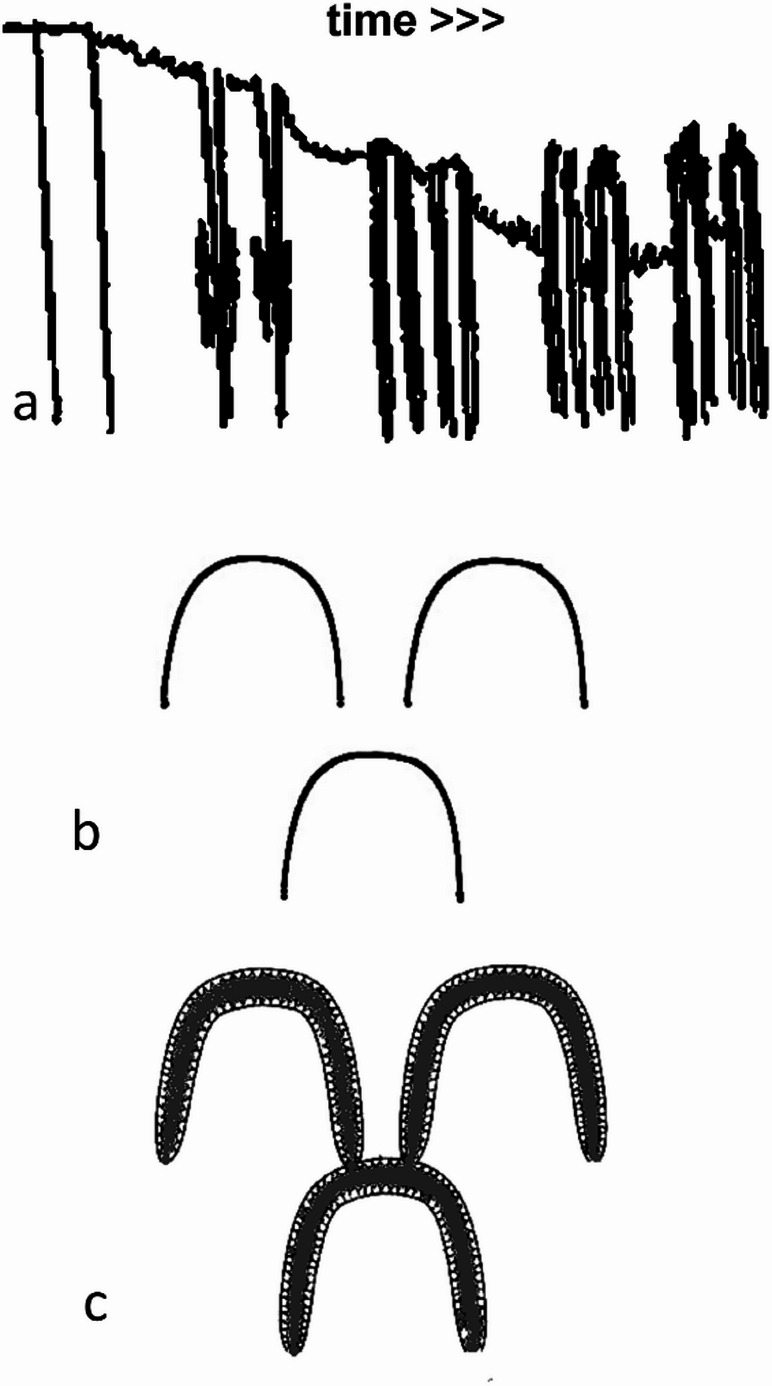
Diagrams showing the sequence of events during acid etching of enamel (and as is shown in the present study in strong peroxide treatments of enamel). **a**. vertical section, time scale left to right, depth scale top to bottom. Prism boundary discontinuity space is expanded and adjacent enamel porosity increased, followed by (re)precipitation of acid calcium phosphate species, which, being less soluble in acid are left proud of the ‘etched surface. **b**. surface-parallel, TS prism view. Starting condition. The black line represents the prism boundary discontinuity space. **c**. same projection, after some etching, the prism boundary discontinuity space has expanded so that boundaries join to make a continuous honeycomb with the adjacent enamel porosity partially infilled with acid-resistant reprecipitate

Acid ‘etching’ enamel is destructive and generates a new structure in depth as it occurs ((Fig. 5a–c: [8, 9, 12]). Dissolution superficially is associated with the precipitation of solubilised calcium and phosphate in the adjacent boundary discontinuity space in deeper layers, which therefore appear relatively brighter in BSE-SEM images. The acid calcium phosphate species (e.g., Ca(H_2_PO_4_)_2_.H_2_O) so formed are selectively resistant to acid dissolution and these regions eventually remain proud of an etched surface, as indicated in the diagrams (Fig. 5a–c and Suppl. Figures 4 and 5). From the BSE-SEM studies reported here, we identify this process as also having occurred in the etching caused by stronger peroxide bleach treatments. What were once under-mineralised, separated, prism boundaries come to have a relatively higher mineral content and become joined together – which is not a normal feature, and can only be explained by demineralisation and later remineralisation within the same tissue. The weaker and less acidic 6% HP gel did not cause this effect even after long treatment periods, indicating that this is a much safer regime.

Indentation hardness testing must displace material. Both reflection LM and SEM images show elevations of enamel surrounding the indent depressions, and this the more so the more softened the enamel.

Can the undesirable effects of bleaching be reversed if not prevented? Many researchers have followed the line of making the bleach neutral or slightly alkaline or adding calcium and phosphate or fluoride to the bleach to suppress demineralisation and such efforts are highly commendable in minimising damage [26, 28, 5, 16, 17, 18, 27, 30, 32, 35, 36, 37, 39, 44, 31]. The damage may self-heal to an extent in vivo due to the uptake of components from saliva [4, 46].

Another predictable effect of all bleaching is the un-glueing of the crystals. Can the original glue be replaced with another? Could such a treatment be tailored to the repair of breaching? One incidental observation in the present study indicates that this may be so. In one instance we found an increase in VH after a bleached section was reglued to the slide with cyanoacrylate. Here superglue could have penetrated the enamel porosity caused by bleaching in these thin sections. Obviously, using cyanoacrylate would be very dangerous in the mouth. The natural recovery of microhardness in vivo [46, 4] might be attributed to either the acquisition of mineral ions or of salivary peptides or proteins. The use of natural and synthetic polypeptides in caries prevention is currently an active research area.

To us, it was a surprising finding that NaOCl bleaching did not reduce the VH: there was even a hint that it might have increased slightly. This would be explained by an increased possibility for the enamel crystallites to fuse by some limited growth at their surfaces after the removal of the intervening enamel protein matrix glue. However, hypochlorite bleach is such bad news for dentine and cementum that we can see no clinical justification for its application to external tooth surfaces.

### Concluding Remarks

Peroxide bleaches not only destroy the residual enamel matrix protein [amelogenin and enamelin etc.] binding the carbonated apatite crystallites together – which is to be expected – but also attack the mineral phase: the higher the peroxide concentration, the lower the pH, the more the damage in depth. Neutralising the acidity will prevent demineralisation, but the bleach will still degrade the intracrystalline organic matrix adhesive which holds the enamel together. Softened, more porous enamel left after peroxide bleaching will be less wear resistant and more prone to staining by food and drink pigments.

The dental trade implies that it is safe for *clinicians* to use 35% peroxide gels whilst the patient should never do so [Anon article ‘Irreversible damage’ caused by illegal tooth whitening treatments, Dentistry Nov/Dec 2025]. These are acidic and damaging.

## Supplementary Information

Below is the link to the electronic supplementary material.

Supplementary Material 1

Supplementary Material 2

Supplementary Material 3
