# Supplementary material for: Enamel and Bleaching or Breaching: Vickers Hardness and Backscattered Electron Imaging
Source: Calcif Tissue Int. 2026 Apr 1;117(1):51. doi: 10.1007/s00223-026-01518-6 (PMC13043528; doi:10.1007/s00223-026-01518-6)
Supplement: Supplementary file 1 — Supplementary Material 1 [file 223_2026_1518_MOESM1_ESM.docx]

**Supplemental statistical output**


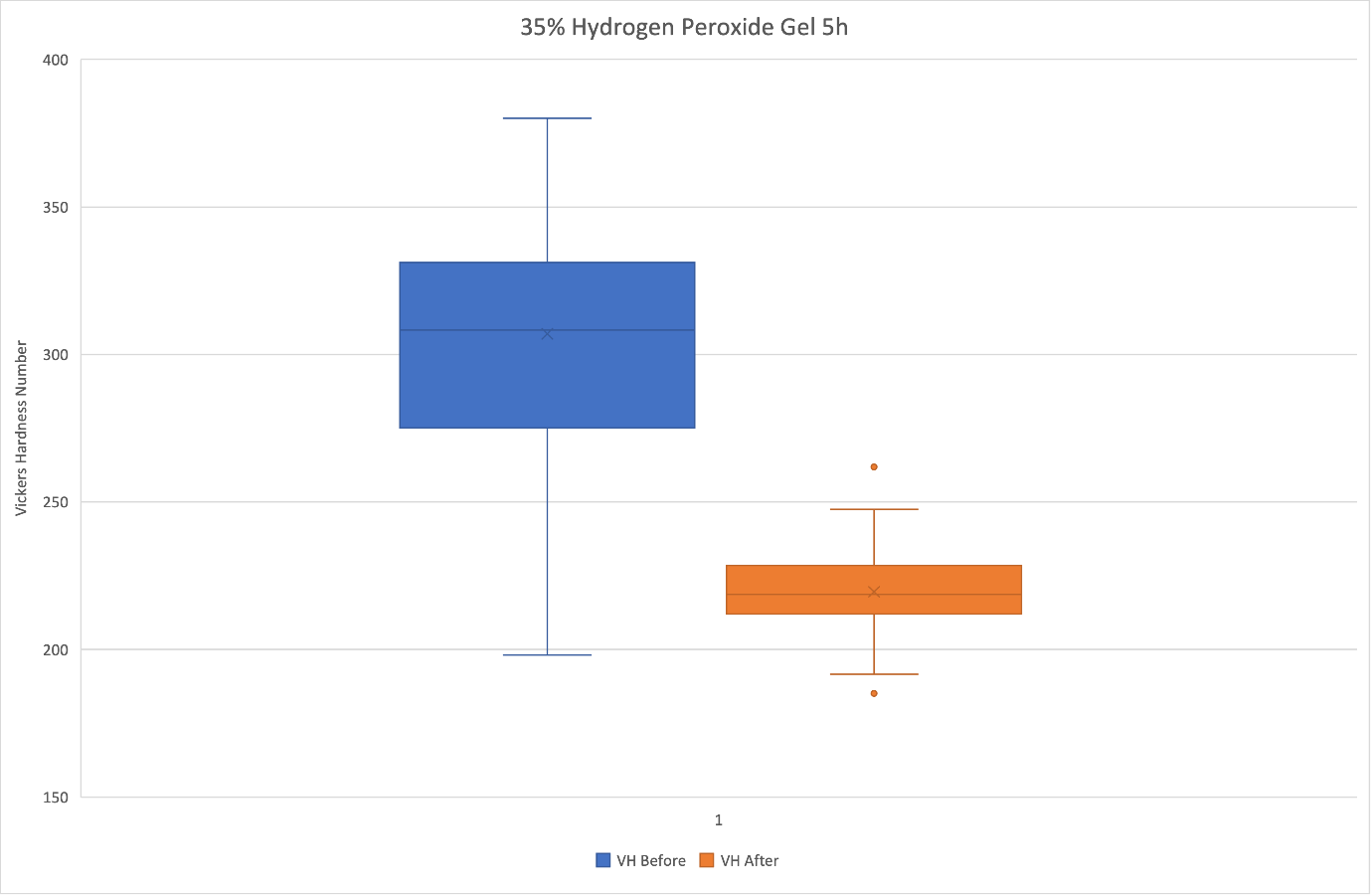


**
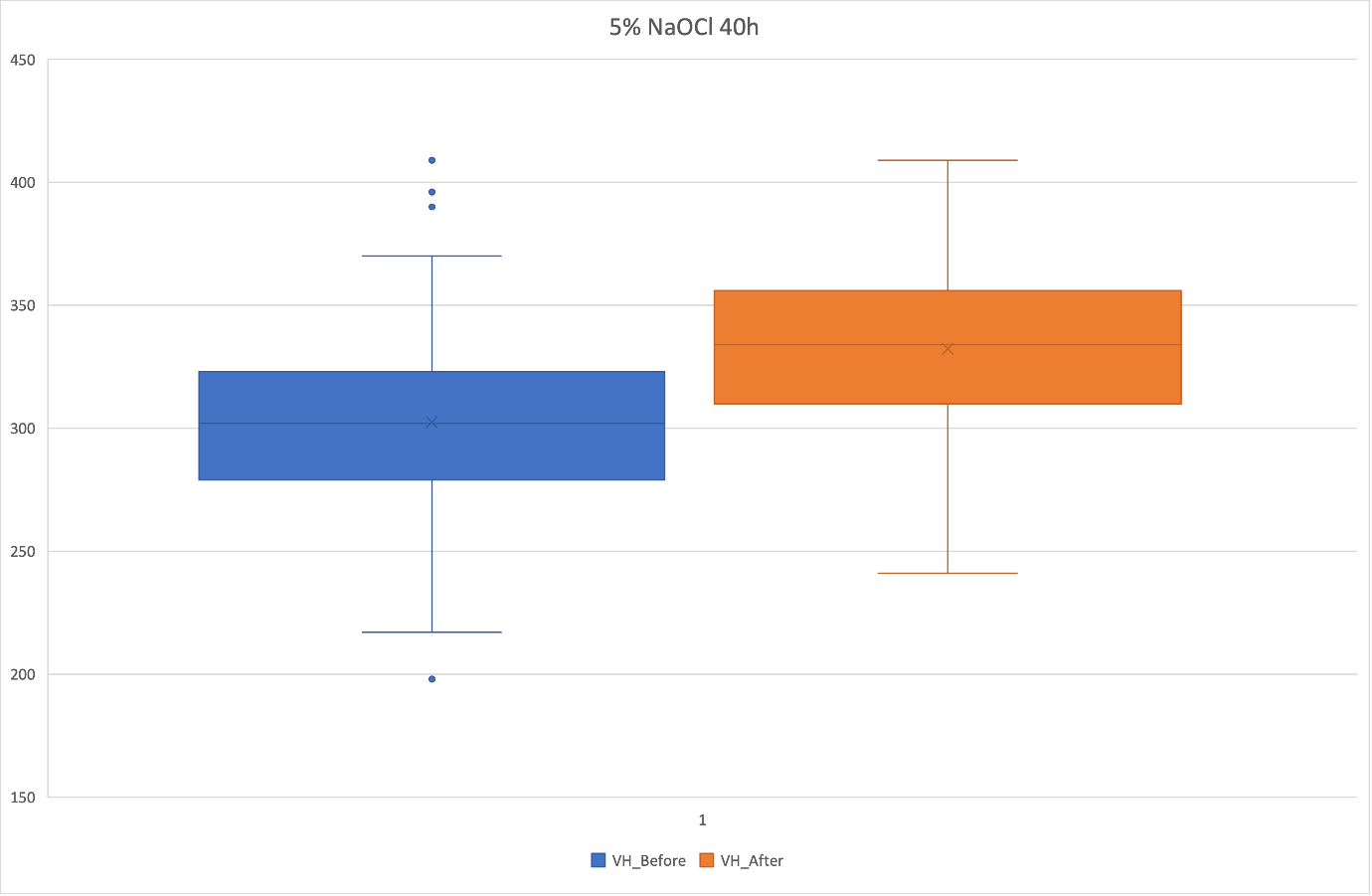
**

**
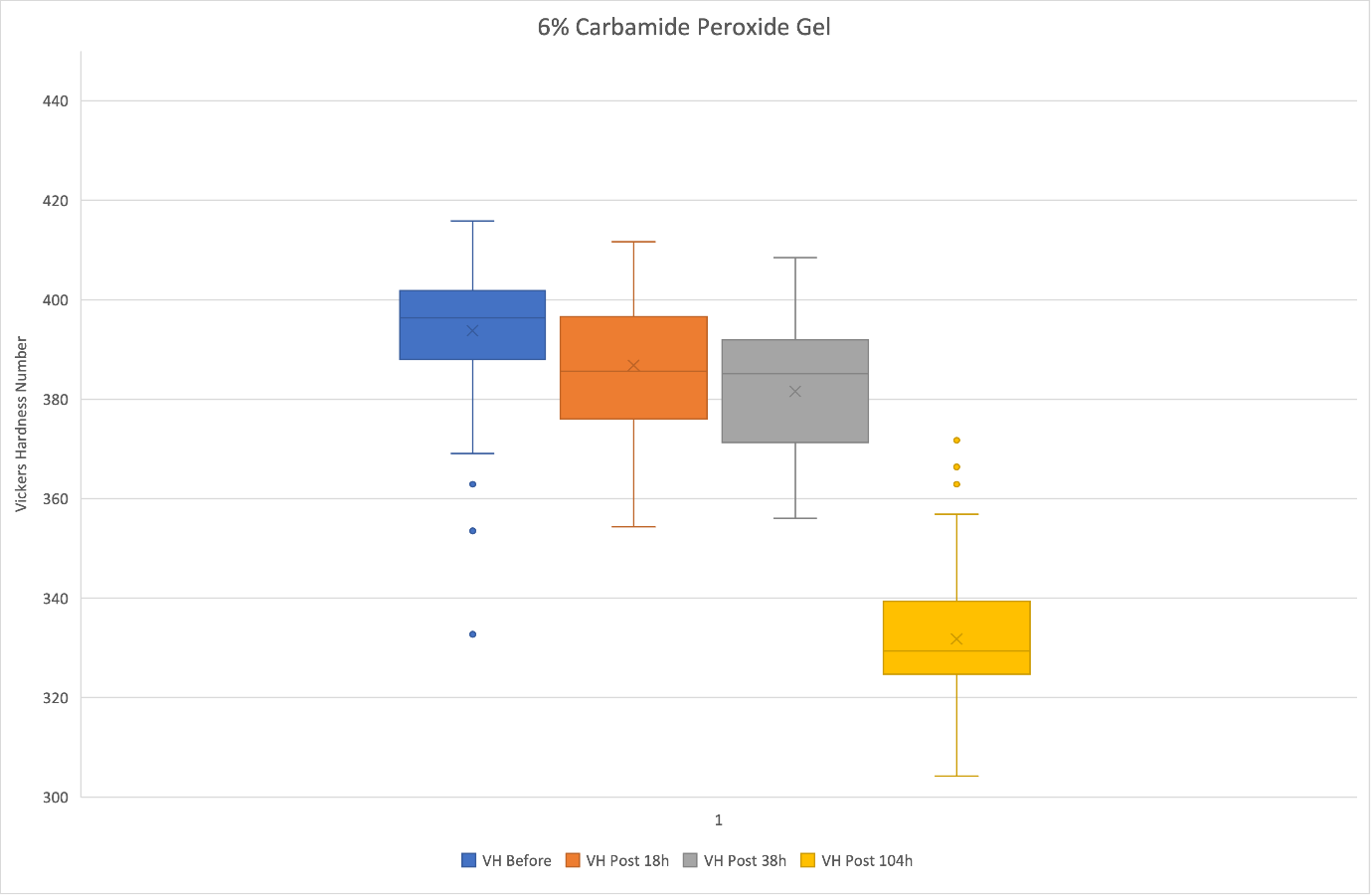
**

**
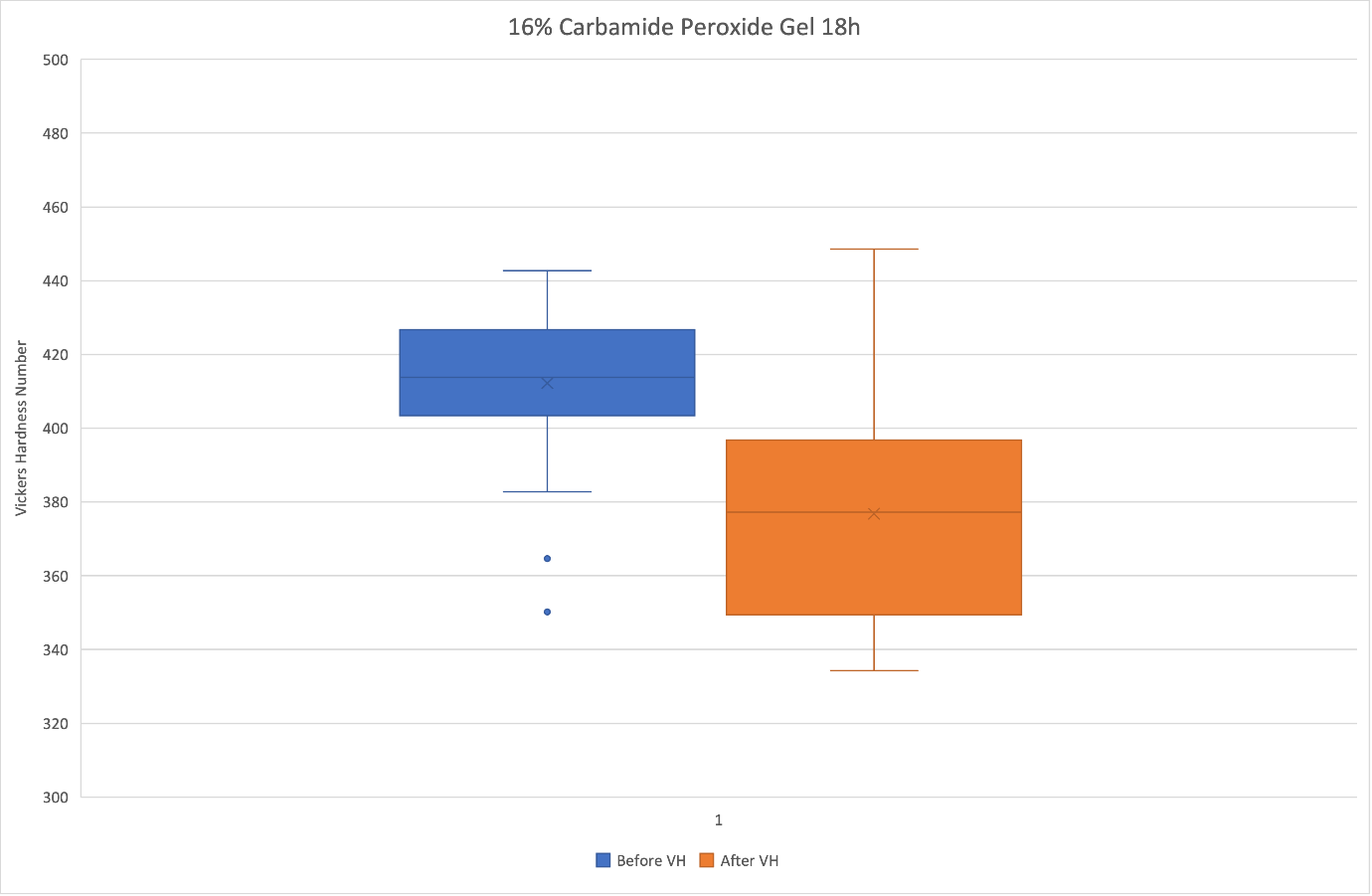
**

**
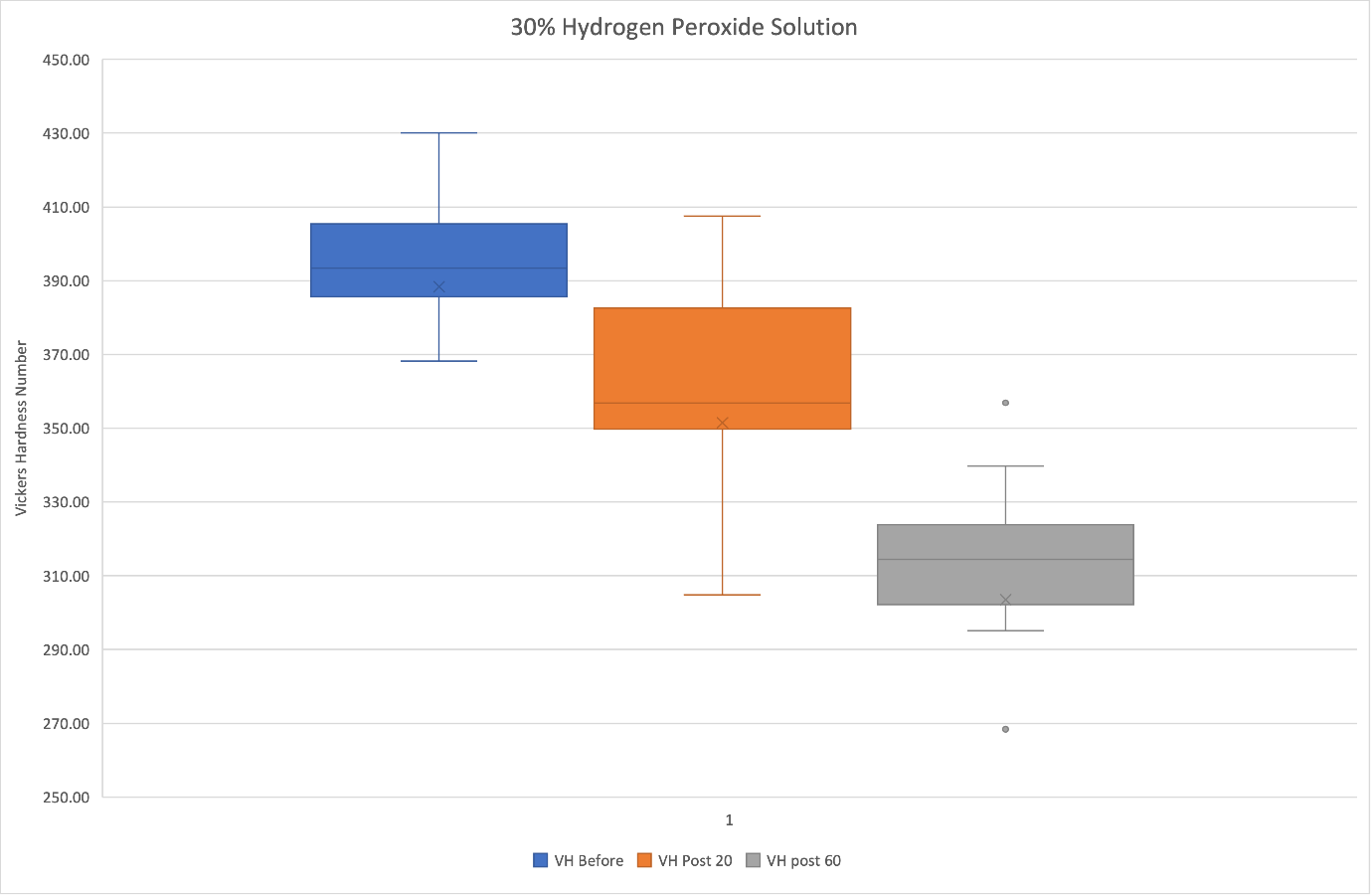
**

**
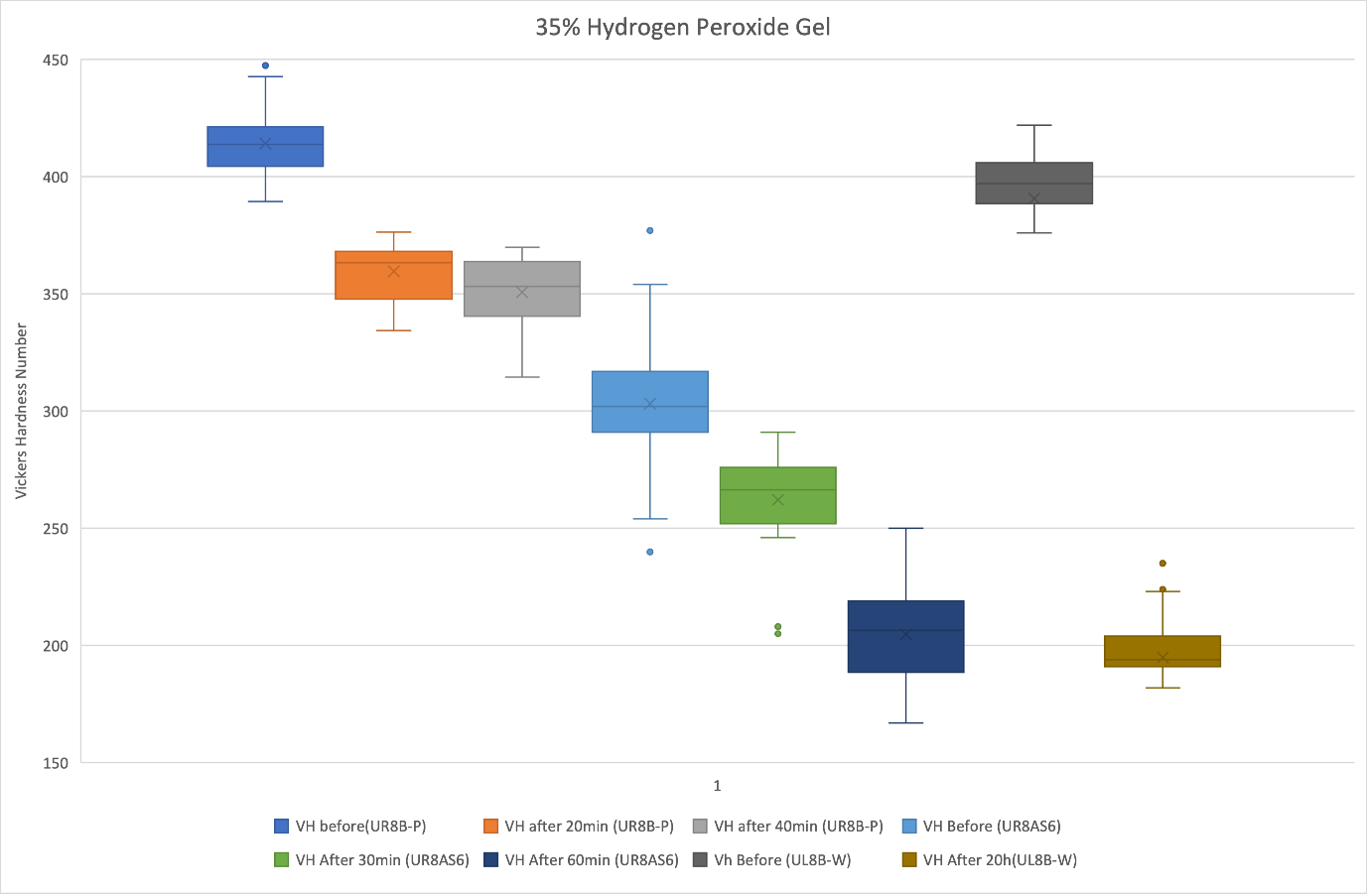
**

3 different experiments/samples reading from left to right.

**
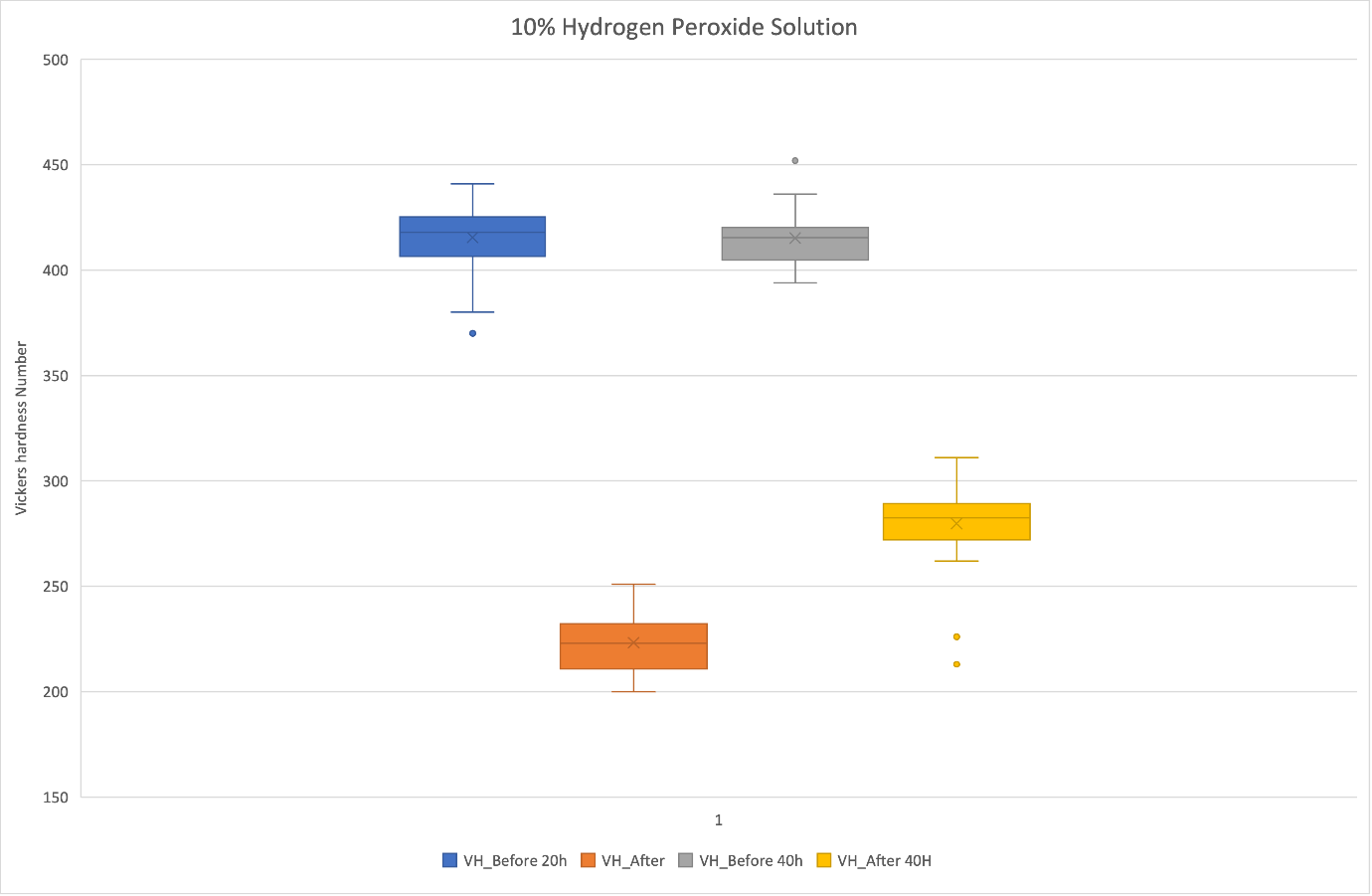
**

**2 different experiments/samples**
