# Supplementary material for: Enamel and Bleaching or Breaching: Vickers Hardness and Backscattered Electron Imaging
Source: Calcif Tissue Int. 2026 Apr 1;117(1):51. doi: 10.1007/s00223-026-01518-6 (PMC13043528; doi:10.1007/s00223-026-01518-6)
Supplement: Supplementary file 2 — Supplementary Material 2 [file 223_2026_1518_MOESM2_ESM.pdf]

SUPPLEMENTARY FIGURES to be submitted in PDF format, no moving image files

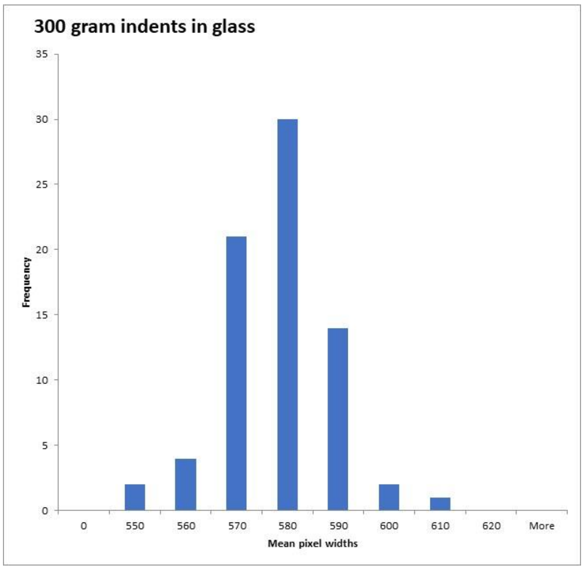

Supplementary Figure 1. There is a wide spread of VH values even for uniform materials, here illustrated with 300 gram indents in microscope slide glass.

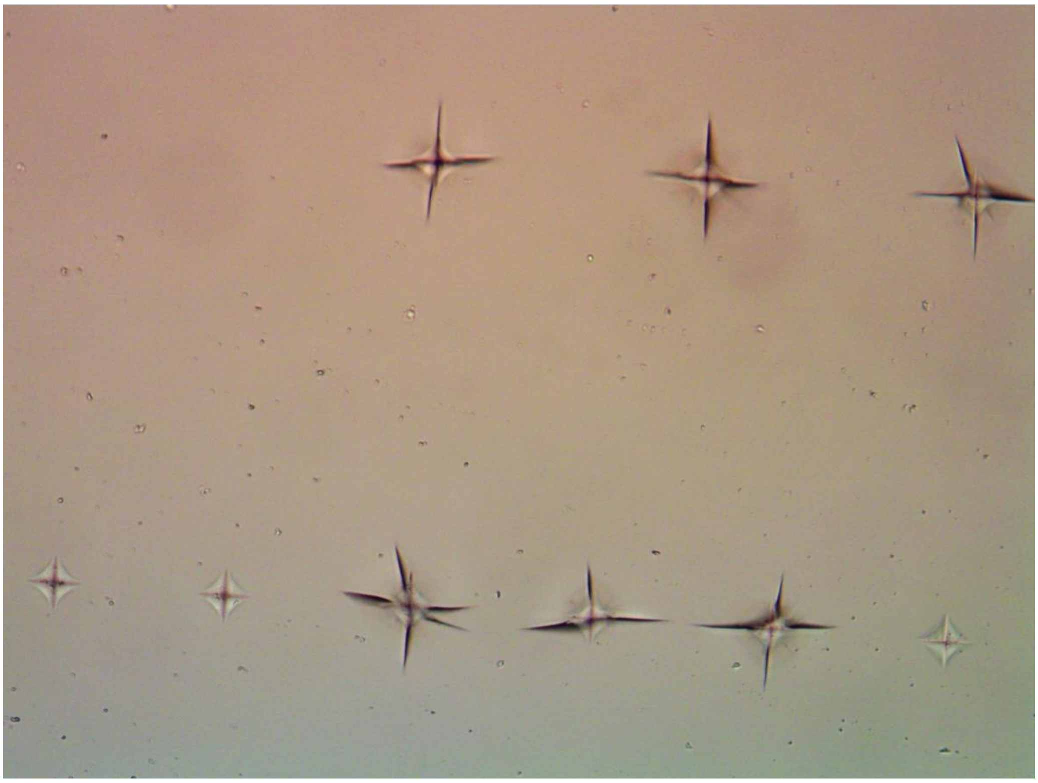

Supplementary Figure 2. Transmitted light image of 300 gram indents in microscope slide glass, part of the field stained with black marker pen ink after cleaning: this reveals extensive cracking beyond the domain of the indents proper. Field width = 570 µm.

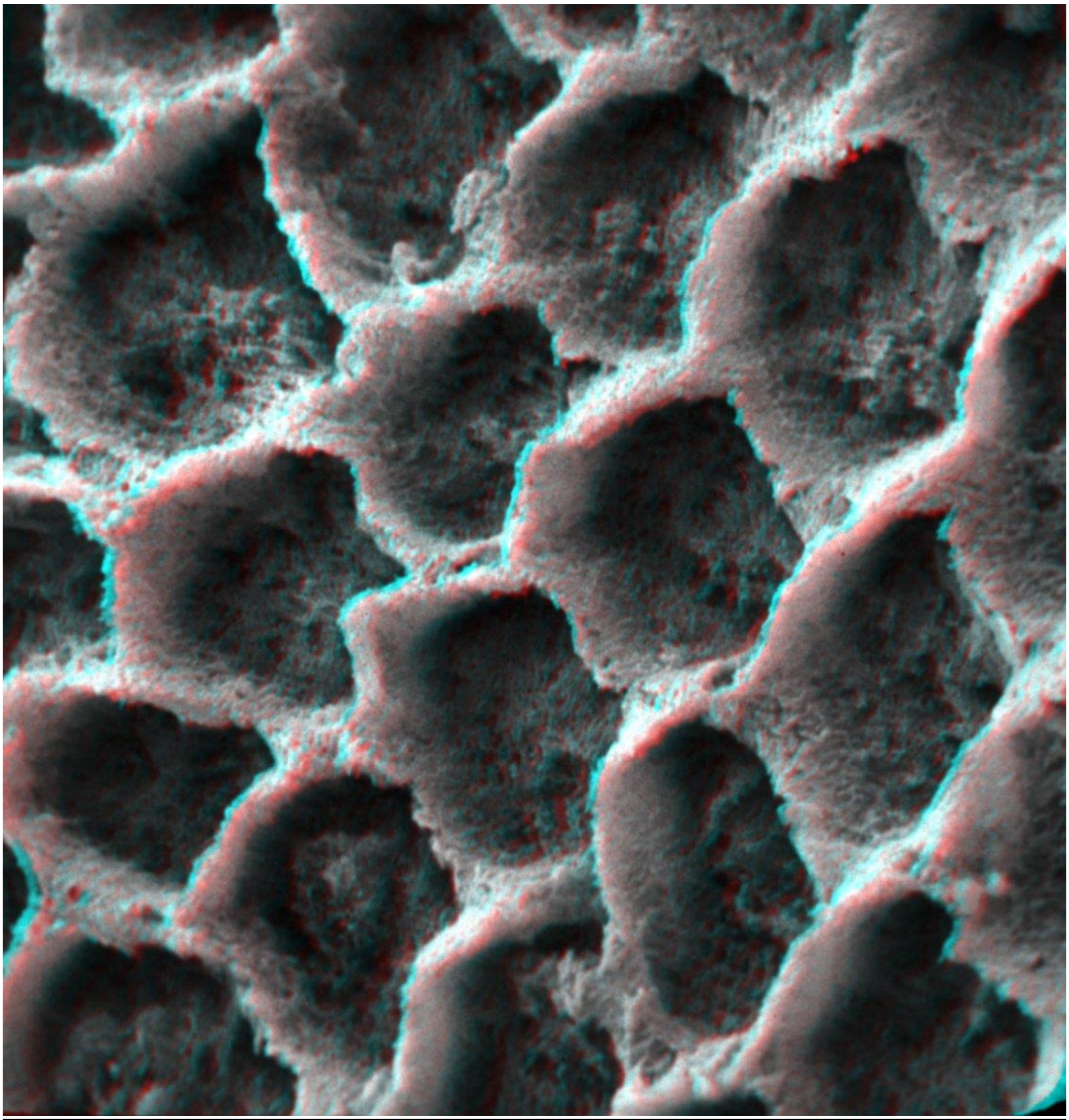

Supplementary Figure 5. Stereo-pair, tilt angle difference 10°, secondary electron SEM image of 5 minutes N/25 hydrochloric acid etched premolar lateral enamel presented as an anaglyph which should be viewed with RED [left eye] CYAN filter spectacles. These images were recorded with a Cambridge Stereoscan S4-10 SEM using secondary electrons, 10kV accelerating voltage from gold coated specimen. Field height = 20 µm.

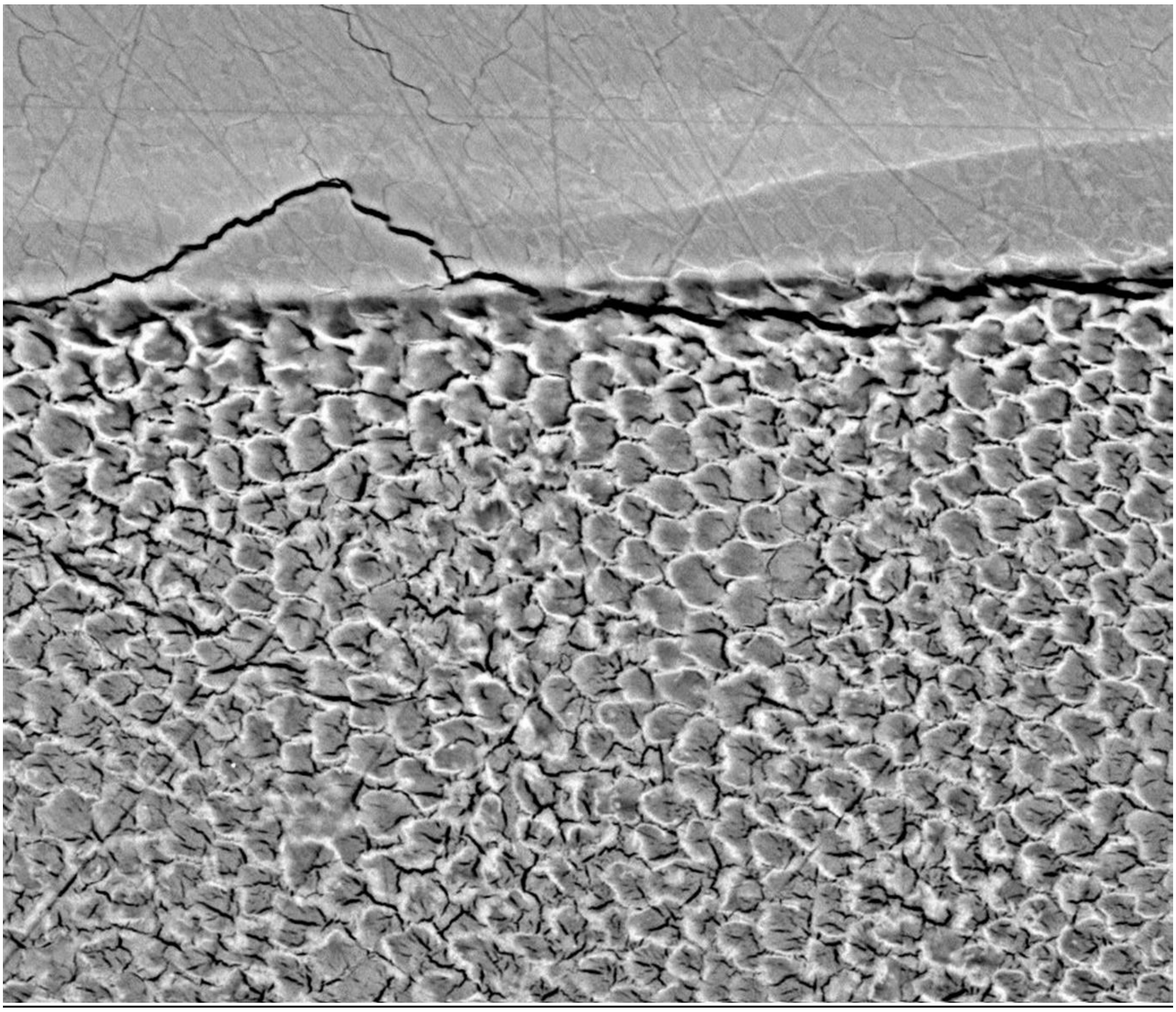

Supplementary Figure 6. Lower third molar, distal, polished surface facet, upper part of field of view was protected with nail varnish, now removed: treated with 30% hydrogen peroxide solution for 66 hours. Surface has eroded leaving etching pattern with continuous elevated honeycomb which was not part of the original enamel microstructure. Field height = 176 µm.

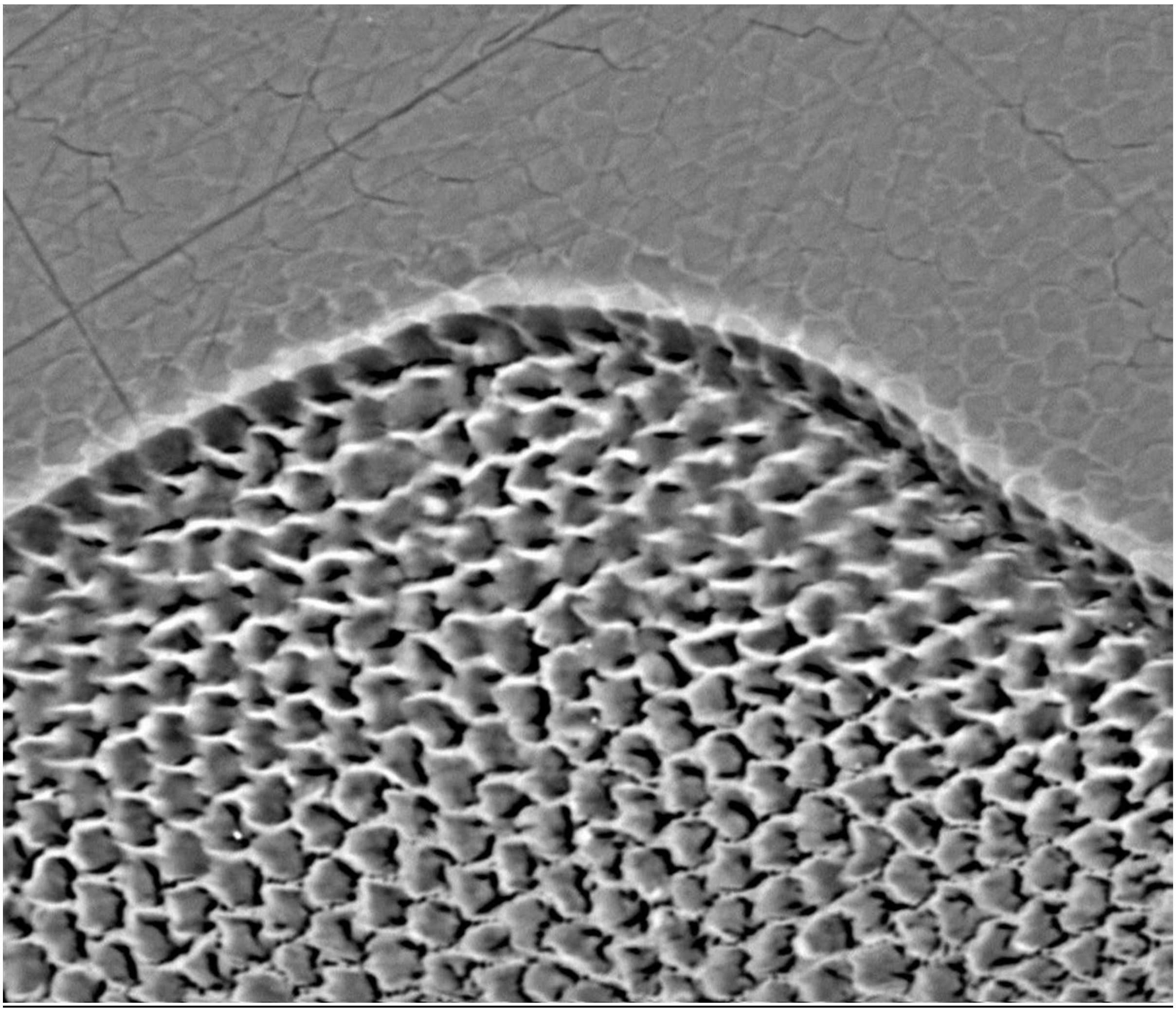

Supplementary Figure 7. Polished surface facet, upper third molar treated with 30% hydrogen peroxide solution for 66 hours: upper part was protected with nail varnish which is now removed: surface has eroded leaving etching pattern with continuous elevated honeycomb. Field height = 144 µm.

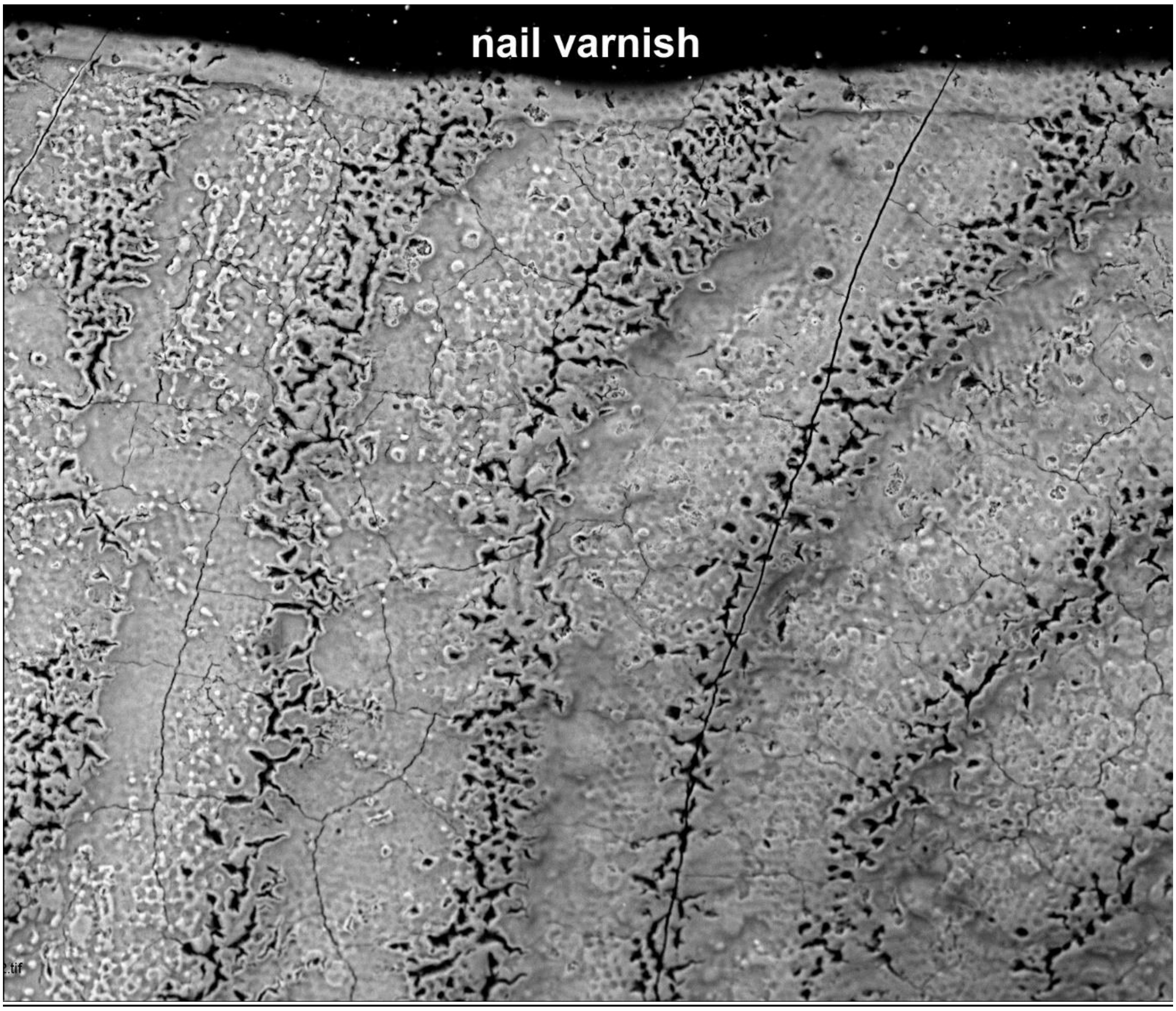

Supplementary Figure 8. Mesial of upper third molar, natural tooth surface, treated with 30% hydrogen peroxide solution for 66 hours: upper part was protected with nail varnish which has shrunk back: the treated surface has eroded and the cracks which are typical signs of early carious lesions have expanded. Field height = 541 µm.

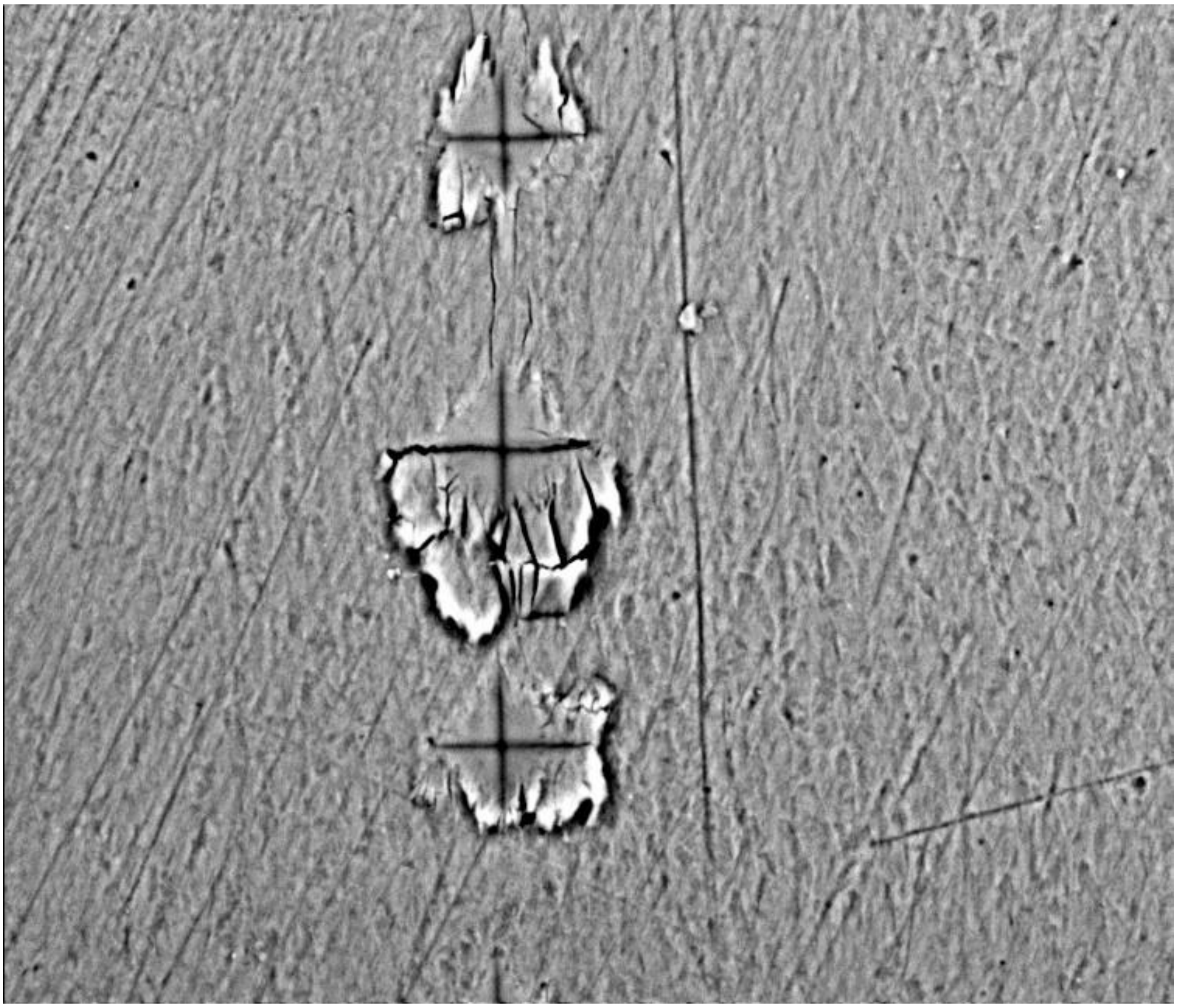

Supplementary Figure 10. 5% NaOCl treated section surface. The tendency to eject material from the indents was higher in the longitudinal sections than in the slab surface samples and in previously 'bleached' samples. Upper third molar. Field height = 166 µm.
