# Supplementary material for: Enamel and Bleaching or Breaching: Vickers Hardness and Backscattered Electron Imaging
Source: Calcif Tissue Int. 2026 Apr 1;117(1):51. doi: 10.1007/s00223-026-01518-6 (PMC13043528; doi:10.1007/s00223-026-01518-6)
Supplement: Supplementary file 3 — Supplementary Material 3 [file 223_2026_1518_MOESM3_ESM.docx]

**SUPPLEMENTARY MOVIE IMAGES** submitted in DOCX format when movies play automatically

**
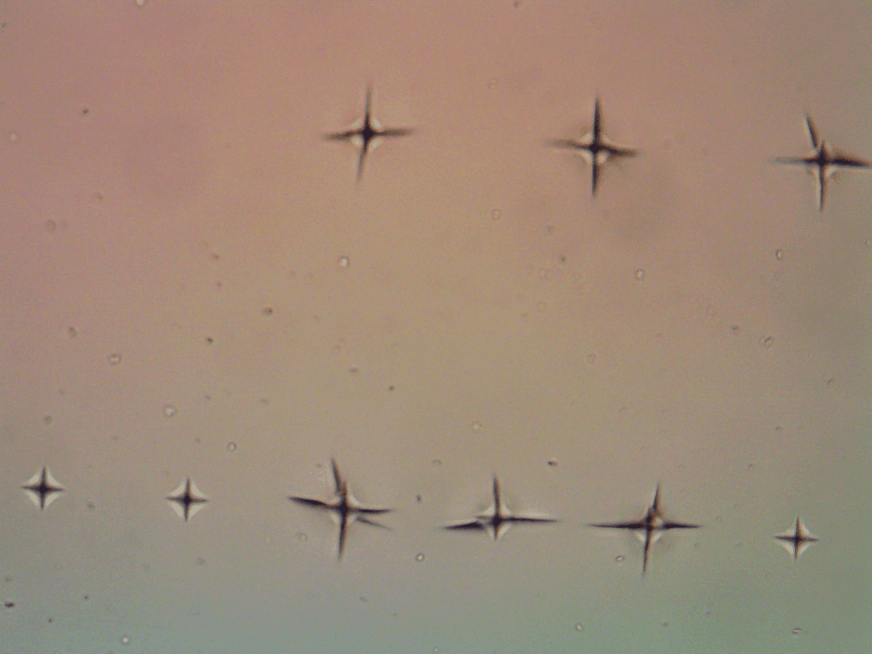
Supplementary Figure 3.** There is a wide spread of VH values even for uniform materials, here illustrated with 300 gram indents in microscope slide glass. Transmitted light image of 300 gram indents in microscope slide glass, part of the field stained with black marker pen ink after cleaning, showing extensive cracking beyond the domain of the indents proper. This is a through focus stack at 2µm intervals presented as a GIF file, which will self-play in DOCX and PPTX file formats and easily viewed with ImageJ-Fiji/Image/Stacks/Animation. Field width = 570 µm.

**
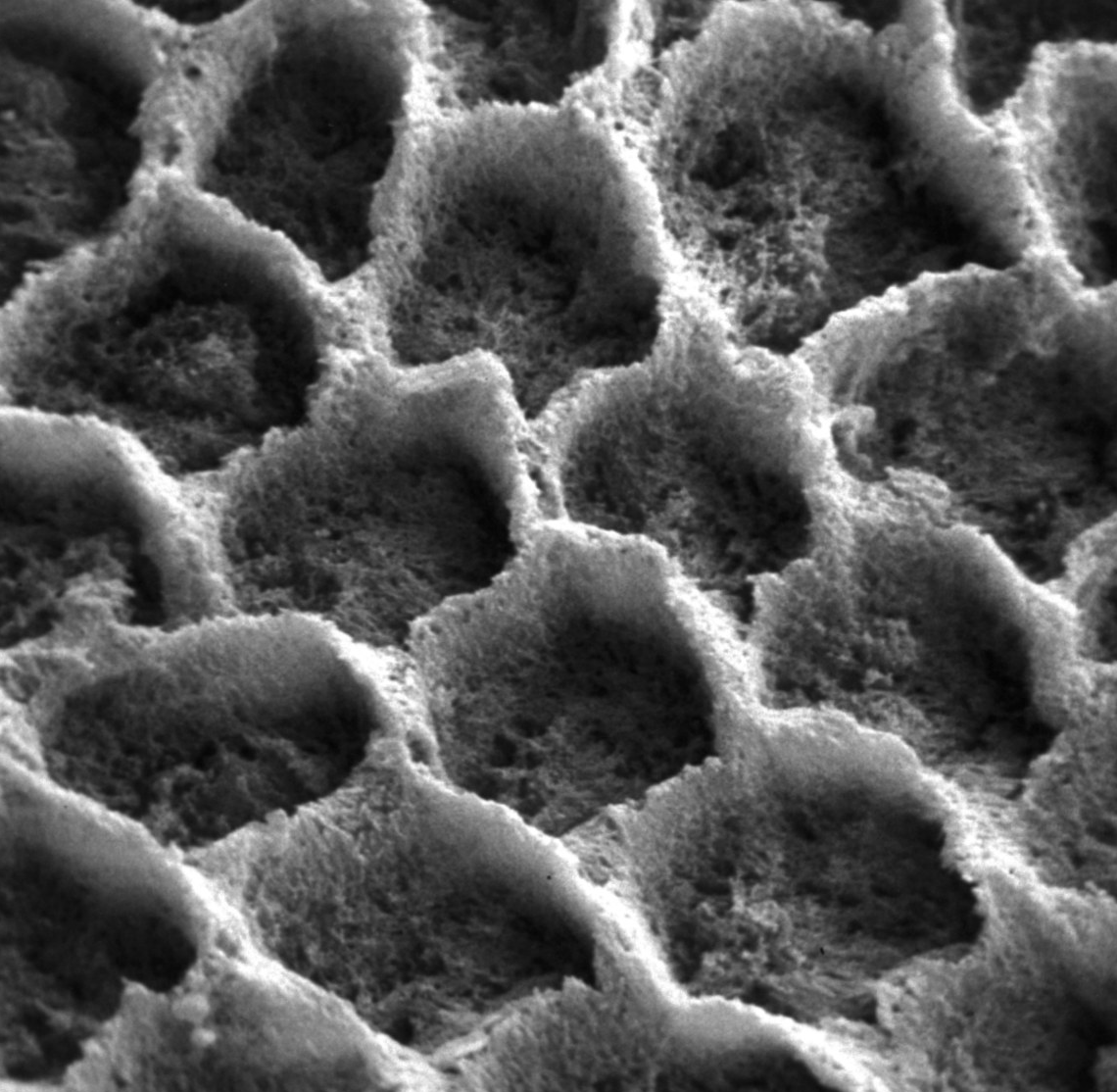
**

**Supplementary Figure 4.** Stereo-pair, tilt angle difference 10°, secondary electron SEM image of 5 minutes N/25 hydrochloric acid etched premolar lateral enamel presented as a GIF file which will play automatically if loaded into a PowerPoint file or can be opened with ImageJ-Fiji/Image/Stacks/Animation. This 3D image shows how a continuous honeycomb structure has developed which was not part of the original enamel microstructure. These images were recorded with a Cambridge Stereoscan S4-10 SEM using secondary electrons, 10kV accelerating voltage from gold coated specimen. Field width = 20 µm.

*
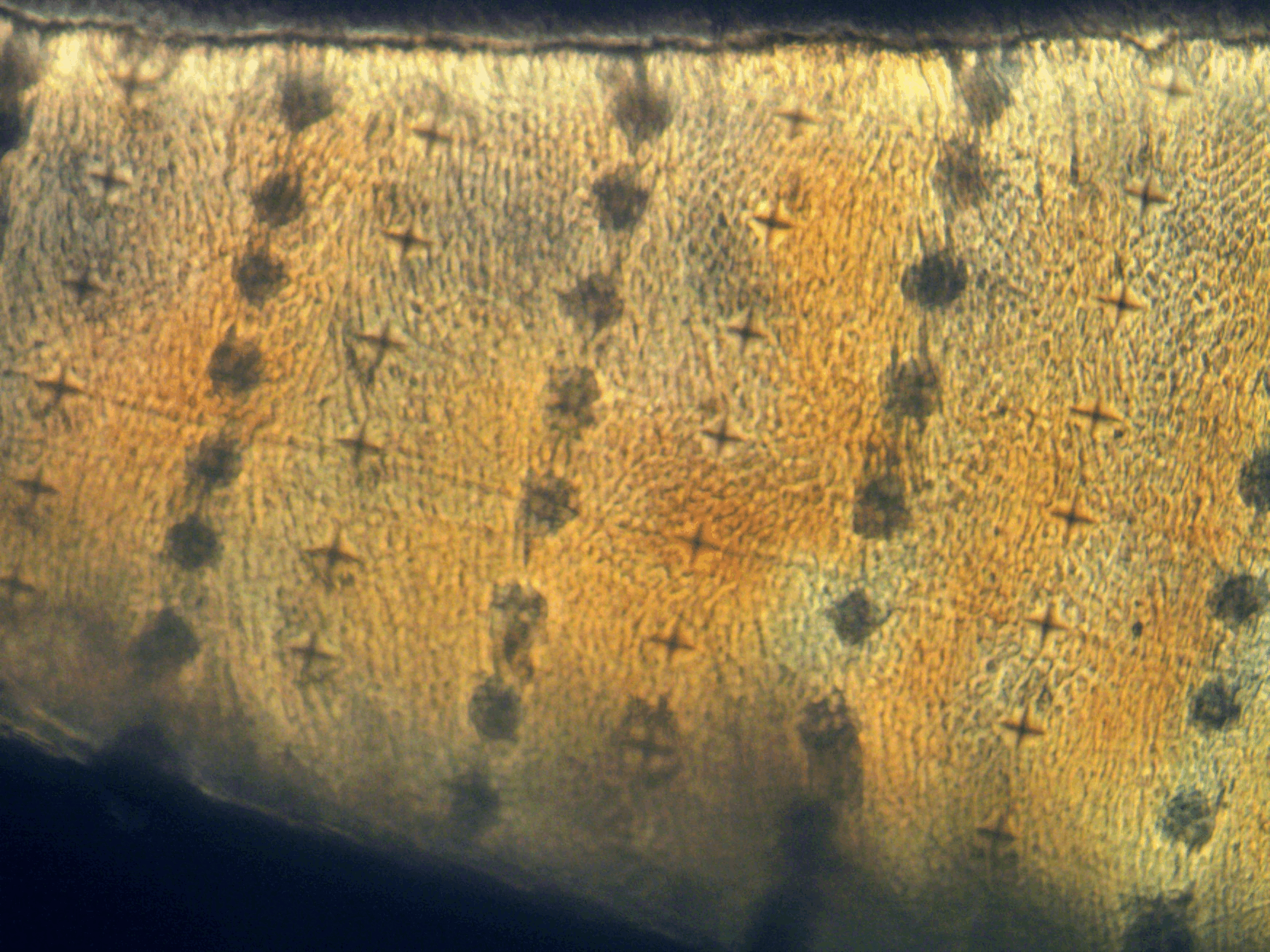
*

**Supplementary Figure 9. Same field as text Fig 1c**. Transmitted circularly polarised light image of lateral enamel in longitudinal section of upper third molar, showing damage zones [dark] around the pre-treatment rows of indents by 35% hydrogen peroxide gel bleaching. Field width = 570 µm. Through focus stack.
